# The modular mind and psychiatry: toward clinical integration with a focus on self-disorders

**DOI:** 10.3389/fpsyg.2025.1570049

**Published:** 2025-04-25

**Authors:** Gheorghe Ilie, Adrian V. Jaeggi

**Affiliations:** Institute of Evolutionary Medicine, University of Zurich, Zurich, Switzerland

**Keywords:** cognitive modularity, psychiatry, self-disorders, enzymatic computation, information encapsulation, modular conflict, classification of mental disorders, evolutionary psychology

## Abstract

One of the foundational tenets of evolutionary psychology, the modular view of the mind, offers promising applications for clinical psychiatry. This perspective conceptualizes the mind as a collection of specialized information-processing modules, shaped by natural selection to address adaptive challenges faced by our ancestors. In this paper, we propose several points of integration between the modularity framework and clinical psychiatric practice. First, we argue that the descriptive psychopathology of self-disorders provides evidence supporting the modular view, demonstrating how a dysfunctional minimal self may expose the mind's modular architecture to conscious awareness. Next, we will explore how the modular perspective can illuminate the nature of intrapsychic conflicts. Finally, we will discuss how evidence from neuropsychiatric syndromes supports the modular view of the mind and, in turn, how this perspective can provide a basis for classifying mental disorders.

## 1 Introduction

In this paper, we explore how the modular view of the mind informs our understanding of neuropsychiatric disorders and, in turn, how these disorders provide empirical support for modularity in the mind. Although the intersection of modularity and psychiatry was elegantly explored by Zielasek and Gaebel (2008, 2009)—laying important groundwork for applying this framework to psychiatric conditions—our approach diverges by centering on ego-pathology, a group of disorders characterized by disruptions in the sense of self. We begin by defining modularity—first in biological terms more broadly (Section 2), and then in cognition more specifically (Section 3). Our discussion focuses on Jerry Fodor's classical concept of modularity in cognition, examining why many of his criteria are unlikely to define a biologically informed modular mind based on current evidence and outlining contemporary views on modularity in evolutionary psychology. Section 4 reinforces this point, cautioning against oversimplified models of cognition. In the following three sections we present the core of our argument. In Section 5 we introduce the concept of self-disorders from classical psychopathology, recognizing both their historical significance and their validity in contemporary research. We then argue that in self-disorders, the modular architecture of the mind is revealed to conscious awareness when the sense of self fails. Using the concept of semantic tagging and the prerequisites of a minimal self, we outline the neurophenomenological link between information processing and the subjective manifestations of self-disorders. At the same time, we theorize how information may be handled by a modular mind in the context of self-disorders, a perspective not previously explored. In Section 6, we present empirical evidence supporting modularity in self-disorders and in Section 7 we propose methods for testing the claims outlined in Section 5. Section 8 then explores information encapsulation in the context of intrapsychic conflict. Finally, Section 9 broadens the scope by demonstrating how evidence from dissociation studies supports a modular architecture of the mind and how, in turn, the modular framework may serve as a valuable organizing principle for classifying neuropsychiatric diseases.

In presenting our arguments, we integrate modularity with elements of embodied cognition and network neuroscience, emphasizing that modularity complements rather than supersedes these perspectives. Modularity can strengthen network-based frameworks (Alcalá-Corona et al., 2021), as we highlight in the context of self-disorders in Section 6. It also explains how distinct neuropsychological functions can be selectively impaired while others remain intact. In contrast, a network perspective shows that disease mechanisms rarely affect a single module in isolation. Instead, disease progression follows network topology, spreading from specific functional modules through hub regions—highly connected nodes within a network—ultimately leading to widespread dysfunction (Seeley et al., 2009; Pievani et al., 2014; Fornito et al., 2015). Embodied cognition integrates with modularity, particularly in bodily ownership disruptions in self-disorders and in melancholic depression, as explored in Section 5. Furthermore, modular decomposition plays a key role in understanding pathophysiology, as identifying disease modules—clusters of functionally related, co-dysregulated genes (Lucchetta and Pellegrini, 2020)—can reveal new disease-associated pathways in various conditions, including temporal lobe epilepsy (Moreira-Filho et al., 2015), pancreatic cancer (Long et al., 2016), and coronary artery disease (Liu et al., 2016). Recognizing the modular architecture of disease can thus advance targeted therapy, while network approaches can identify vulnerable hubs (e.g., in neurodegeneration) and guide early interventions to slow disease progression (Zhou et al., 2012). Thus, modularity, embodied cognition, and network neuroscience—each operating at distinct yet complementary levels of analysis—together provide a more comprehensive framework for understanding cognitive function and dysfunction.

## 2 Modularity as a general concept

Modularity holds different meanings across disciplines (Zelditch and Goswami, 2021). However, in its widest scope, it refers to interacting, functionally specialized, and semi-independent units—a concept ubiquitous across the biological world at multiple scales (Hartwell et al., 1999; Huitzil and Huepe, 2024). Biological systems are decomposable into such discrete functional units, spanning levels from the molecular to the ecosystemic (Huitzil and Huepe, 2024). At the molecular level, examples include cell cycle protein complexes (John et al., 2001) and gene regulatory networks (Davidson and Erwin, 2006; Wellik, 2007). At the cellular level, metabolic networks (Jeong et al., 2000; Ravasz et al., 2002) and cellular interaction networks (Barabasi and Oltvai, 2004; Qi and Ge, 2006) provide additional instances. This principle extends to higher levels of organization, such as tissues, organs, and even ecosystems (Solé and Montoya, 2001; Montoya et al., 2006). Modularity enables systems to repurpose and recombine existing components, fostering innovation and adaptability in response to environmental shifts. It also helps contain disruptions, allowing subsystems to evolve independently while improving both the efficiency of information processing and system-wide integration. Furthermore, by structuring simple elements into more complex arrangements, modularity facilitates the emergence of new functions and supports specialization within complex systems (Huitzil and Huepe, 2024).

Within modules, elements are more interconnected with each other than with neighboring modules (Cheverud, 1996; Wagner, 1996; Hartwell et al., 1999; Von Dassow and Munro, 1999; Debat et al., 2000; Newman, 2006; Klingenberg, 2008). This principle also applies to brain organization, where clusters of neurons exhibit dense intramodular connectivity and relatively sparse connections to neighboring modules (Sporns et al., 2004, 2005; Meunier et al., 2010; Gazzaniga, 2018; Gazzaniga et al., 2019). Functional specialization is their hallmark (Passingham et al., 2002; Bullmore and Sporns, 2012; Petersen and Sporns, 2015) as evidenced by their consistent activation during specific tasks (Kanwisher, 2010), such as the fusiform face area being reliably activated during face perception tasks (Kanwisher et al., 1997; Kanwisher and Yovel, 2006).

## 3 Modularity in cognition

The existence of mental modules—cognitive mechanisms specialized for processing specific types of information—makes sense from an evolutionary perspective, as natural selection is expected to shape mechanisms that are well-suited to particular tasks (e.g., Burkart et al., 2017); as such the concept has been widely adopted by evolutionary psychologists (Tooby and Cosmides, 1992; Barrett and Kurzban, 2006; Carruthers, 2006) The concept of mental modularity was first introduced to cognitive science by Jerry Fodor in his 1983 book, *The Modularity of Mind*, where he proposed a set of a priori properties that mental modules are likely to possess. These include, among others, domain specificity (processing specific types of information, p. 47–52), automaticity and rapid information processing (p. 61–64), informational encapsulation (knowledge in other domains doesn't influence their operations, p. 64–86), shallow outputs (lacking broader contextual and interpretive depth, p. 86–97), fixed neural architecture (p. 98–99), specific breakdown patterns (damage results in localized deficits, p. 99–100) and characteristic ontogeny (developing in a uniform manner across individuals, p. 100–101) (Fodor, 1983).

Fodor's criteria have faced extensive criticism, including from evolutionary psychologists, for their biological implausibility and their inability to account for the diversity of evolved mental modules (Barrett, 2005, 2007; Pinker, 2005; Barrett and Kurzban, 2006; Barrett et al., 2006). Critics of the modular view, such as Bolhuis et al. (2011), argue that the classical criteria—particularly strict domain specificity and informational encapsulation—are inconsistent with empirical findings from neuroscience, genetics, and developmental psychology (Bolhuis et al., 2011). While certain systems, such as those involved in face recognition, are indeed domain-specific and process a single type of input (e.g., Kanwisher et al., 1997), other systems are expected to integrate inputs from multiple domains to address complex challenges. For example, threat identification likely involves the interaction of motion detection, memory, emotional processing, and motor systems. This view aligns with Bolhuis et al.'s (2011) emphasis on cognitive plasticity, extensive interconnectivity, and feedback loops in the brain, which facilitate the integration of information across multiple domains and support adaptability to novel contexts.

Moreover, informational encapsulation is not an all-or-nothing characteristic of cognitive systems, because few, if any, processes operate in complete isolation (see also Section 8). Instead, cognitive modules often interact in structured ways, promoting cognitive flexibility in solving adaptive challenges (Barrett, 2005). Even ostensibly “peripheral” processes, like visual perception, are modulated by context, personal expectations, and higher-order cognition (Goldstone and Barsalou, 1998; Pylyshyn, 1999; Ditzinger, 2021), illustrating how Fodor's concept of encapsulation applies primarily at the intentional level—reflecting subjective experience—rather than at the functional level, which concerns how cognitive systems actually operate (Pietraszewski and Wertz, 2022). Furthermore, not all cognitive systems are designed to function automatically or process information rapidly, as highlighted by the distinction between System 1 and System 2 (Kahneman, 2011). Pietraszewski and Wertz (2022) argue that automaticity is meaningful primarily at the intentional level—where cognitive processes appear effortless or reflexive—whereas at the functional level, cognitive mechanisms vary in flexibility and deliberation depending on adaptive demands, rather than being inherently automatic (Pietraszewski and Wertz, 2022). Nor do mental modules necessarily have a fixed neural architecture as they are often widely distributed across the brain (Pinker, 2015). For instance, some authors propose that Theory of Mind arises from the interaction of the 3 large-scale neural networks—default-mode network, salience network, and frontoparietal network (Ryan et al., 2017), while others argue that it emerges from the activity of 5 specific brain regions (Saxe, 2006).

The properties of mental modules, it has been argued, are a matter of empirical discovery rather than predefined assumptions (Sperber, 1994). A major source of confusion arises from the conflation of modern conceptions of modularity with classic Fodorian modularity (Pietraszewski and Wertz, 2022). Indeed, several alternative non-Fodorian models of cognition have been proposed (Barrett, 2005; Carruthers, 2008). Sperber highlighted a paradox in the title of Fodor's book, *The Modularity of Mind*, noting that Fodor restricted modularity to the “periphery” of the mind—sensory systems like perception—while attributing the “core” processes, such as reasoning, inference, problem-solving, judgment, and decision-making, to general-purpose mechanisms, which he considered non-modular (Sperber, 1994; Barrett, 2015, p. 265). The concept of general-purpose cognitive mechanisms has been widely criticized, particularly by the adaptationist program in evolutionary psychology, which argues that the entire mind, not just its periphery, is modular (Tooby and Cosmides, 1992). Critics invoke several key arguments against general-purpose mechanisms, including functional incompatibility (the inability of a single mechanism to simultaneously optimize behavior across conflicting adaptive challenges), combinatorial explosion (overwhelm caused by infinite possibilities for action, making decision-making infeasible), and a lack of efficacy in clueless environments (insufficient information to deduce effective actions without prior knowledge or specialized mechanisms) (Tooby and Cosmides, 1992; Ermer et al., 2007). However, Pietraszewski and Wertz (2022) caution against strictly categorizing cognitive mechanisms as domain-specific or domain-general, arguing that functional specialization exists on a continuum, with mechanisms varying in specificity and flexibility based on adaptive demands. They emphasize that domain specificity is best understood at the functional level, where mechanisms process particular input classes, rather than as an absolute distinction implying strict encapsulation or independence from other processes (Pietraszewski and Wertz, 2022). A more useful way to address this dichotomy is provided by Sperber (1994), who distinguishes between a module's proper domain—the type of information it evolved to process—and its actual domain, which includes the range of inputs it currently processes, even if they were not part of its original evolutionary function (Sperber, 1994). For example, the face recognition system evolved to process human faces (its proper domain) but can also be activated by face-like stimuli, including caricatures, objects resembling faces, and even schematic face-like patterns (its actual domain) (Johnson, 2005; Tsao and Livingstone, 2008; Hadjikhani et al., 2009; Liu et al., 2014).

According to the adaptationist framework, modules in the mind are specialized information-processing devices shaped by natural selection to solve recurrent adaptive problems encountered by our human ancestors (Tooby and Cosmides, 1992; Buss, 1995; Carruthers, 2006; Barrett, 2015; Pinker, 2015; Lewis et al., 2020). An adaptive problem—defined as a challenge affecting survival or reproduction—can be identified by applying natural selection principles to behaviors such as food acquisition, mating, kin care, cooperation, and aggression (Buss, 2019). This perspective underpins the concept of mental modularity used in this paper, emphasizing functional specialization as its core feature, akin to other evolved biological mechanisms (Tooby and Cosmides, 1992; Barrett and Kurzban, 2006; Carruthers, 2006).

In the current understanding of evolutionary psychology then, a mental module consists of three primary components: an input subsystem that receives informational cues from internal or external sources (e.g., physiological, social, or ecological signals); a processing mechanism that applies algorithms, decision rules, or other computational operations; and an output subsystem that generates behavioral responses, physiological reactions, or further inputs for other modules (Buss, 1991, 1995, 2019; Lewis et al., 2020). For the module to efficiently solve an adaptive problem, its front end must align closely with environmental features (Tooby and Cosmides, 1992). This fit between form and function is the essence of functional specialization, where “form” refers to the information-processing design features of the mechanism (Barrett, 2009). These features work by detecting specific informational cues from the environment or the organism that signal an adaptive problem, activating the system (Carruthers, 2006, p. 7) and informing the organism of the challenge it faces (Buss, 1991, 1995, 2019). Modules also address adaptive problems using inherent assumptions about the world's structure, known as intuitive ontologies, which guide behavior and organize knowledge (Cosmides and Tooby, 1997; Carey, 2009; Boyer and Barrett, 2015). The diversity of adaptive challenges faced by humans suggests a corresponding array of specialized mental modules (Buss, 1991, 1995, 2019). This proliferation of modules is sometimes referred to as the “multimodular mind” (Cosmides and Tooby, 2013) or the “Massive Modularity Hypothesis” (Carruthers, 2006).

In conclusion, although cognitive modularity remains a topic of active debate—either because its conceptual evolution has been underrecognized or because its modern iteration is still conflated with the older Fodorian model—key aspects of its modern form are particularly useful for understanding psychiatric phenomena, as explored throughout this paper. Functional specialization is especially relevant in lesion studies, while intuitive ontologies and the tight form-function fit help explain semantic deficits, which track fitness-relevant environmental elements (e.g., plants, animals, motion detection) (Section 9). Crucially, the diversity and numerosity of cognitive modules align with insights from the psychopathology of self-disorders, as explored in Section 5.

## 4 Words of caution

The above observations about cognitive architecture are necessarily simplified and may reflect our natural tendency to categorize reality. As Nesse cautions, it is essential to avoid the trap of tacit creationism—the implicit view of bodies as engineered machines—by recognizing their full biological complexity (Nesse, 2020, 2022). Bodies are not machines with distinct components designed to perform isolated functions; instead, their interlocked parts carry out overlapping functions, shaped by natural selection to enhance gene transmission. For instance, functions such as fighting infections are distributed across multiple components (Nesse, 2020). Similarly, caution is needed when interpreting the mind through the “mind as a computer” metaphor on which Fodor's classical modularity is based. While Marr's tripartite computational framework (inputs, computational rules, and outputs) remains central to cognitive science (Marr, 1982; Brase, 2014, 2021), and can be applied to evolved cognitive modules, extending the metaphor beyond its source domain can lead to biologically improbable conclusions (Brase, 2002). Instead, “we should be prepared to let the brain inform us about how it solves problems, rather than deciding in advance” (Barrett, 2007).

The organic complexity of brains further resists the oversimplification of mapping one module to one function or one ontological category. While modules can theoretically map onto functions in various ways—one to one, one to many, many to one, or many to many (Barrett, 2020a), their precise alignments remain ultimately empirical questions. This complexity reflects the evolutionary history of human cognitive abilities, which have emerged through hierarchical evolution driven by descent with modification, duplication, and divergence (Barrett, 2012, 2017). These processes have repurposed and specialized ancient mechanisms, creating a brain that integrates ancestral systems with novel adaptations unique to humans (Barrett, 2012, 2017). For instance, mindreading builds on older mechanisms like gaze detection to make sophisticated inferences about the mental states of others (Baron-Cohen, 1997). These cognitive abilities have also been shaped through reciprocal interactions with human-designed environments and tools, leading to further adaptive changes (Barrett, 2012, 2017). Neural sharing of components leads to overlapping and indistinct functional boundaries (Nesse, 2004, 2020, 2022; Anderson, 2010). This underscores that modules are not atomic entities parsing reality into distinct classical categories (e.g., plants, animals, and objects) (Carruthers, 2006, p. 62; Boyer and Barrett, 2015). Instead, they transcend traditional ontological boundaries, addressing fitness challenges arising from interwoven situations in the evolutionary past (Nesse, 1990a, 2022; Boyer and Barrett, 2015). For instance, the behavioral immune system, a suite of behaviors aimed at avoiding infection, activates regardless of whether the pathogenic threat originates from contaminated food, bodily products, infected animals, or humans (Schaller, 2015). This suggests that mental modules respond to specific eliciting features within a category, rather than the category as a whole (Boyer and Barrett, 2015).

Modules are not expected to develop in a fixed, predetermined manner across all individuals. Instead, they emerge in each generation as genes interact with developmental processes and environmental inputs. This process, termed “design reincarnation” (Tooby et al., 2003; Barrett, 2006), highlights the universality of our cognitive architecture (types) alongside individual variation (tokens). Types refer to universally evolved cognitive mechanisms, such as the capacity to recognize faces or acquire language, which have been shaped by natural selection to address adaptive problems. In contrast, tokens represent individual manifestations of these mechanisms, shaped by personal experiences and environmental inputs, like recognizing a familiar face or speaking a specific language (Barrett, 2006, 2012, 2017). This relationship showcases that our species-specific cognitive mechanisms generate flexible responses contingent upon their different inputs (Lewis et al., 2020).

Transcultural research, for example, reveals how environmental variability shapes diverse individual and cultural outcomes. In regions with high pathogen prevalence, for instance, individuals tend to prioritize physical attractiveness in mate selection, because visible traits can signal health and genetic fitness, which become particularly important in disease-prone environments (Gangestad and Buss, 1993). Under these conditions, women tend to prefer more masculine male faces, a relationship that persists regardless of women's specific mating strategies or economic wealth (DeBruine et al., 2010). These environments are also associated with increased collectivism, a trait that can help mitigate disease transmission (Fincher et al., 2008), and tend to foster lower average levels of extraversion and sociosexuality, likely due to the risks posed by increased interpersonal contact (Schaller and Murray, 2008). The influence of cultural and environmental factors extends beyond mating strategies and personality traits to basic senses, such as olfaction. For instance, speakers of Jahai in the Malay Peninsula exhibit remarkable precision in labeling odors using culturally specific terms, in stark contrast to the difficulty English speakers often face in describing smells (Majid and Burenhult, 2014). Similarly, the Maniq language, spoken by a group of hunter-gatherers in southern Thailand, contains numerous odor-specific words that enable detailed categorization and recognition of smells (Wnuk and Majid, 2014). These linguistic differences align with the ecological environments of these communities, as their foraging lifestyle likely necessitates heightened olfactory acuity, which their languages facilitate. This interaction between universal, species-specific mechanisms and environmental and cultural factors produces individualized outcomes, demonstrating developmental plasticity, wherein consistent cognitive types adapt flexibly to varied contexts to generate specific tokens (Barrett, 2020b).

## 5 The unitary self shattered into a thousand pieces

In the following section, we will argue that the descriptive psychopathology of self-disorders supports the multimodular view of the mind described in Section 2. When the pervasive sense of self that typically unifies mental processes breaks down, as it does in self-disorders, the modular architecture of the mind becomes internally exposed to the individual. This disruption reveals the mind's inherent multiplicity, as the lack of synchrony between its components forces the subject into a distressing awareness of their fragmented internal state.

### 5.1 The disordered self

Historically, the concept of “self” has been the subject of numerous philosophical interpretations (Zahavi, 2003). Berrios and Marková (2003) describe how this term entered psychiatric discourse in the 19^th^ century, ultimately contributing to the concept of the “disordered self.” However, they argue that the very notion of the self is merely a metaphor (Berrios and Marková, 2003). By contrast, psychiatrists with a phenomenological perspective reject the notion of the self as either a metaphor or a social construct, viewing it instead as a fundamental aspect of mental life. From this perspective, the self is understood through the lens of “first-personal givenness”—a pre-reflective self-awareness that imbues all experiences with a sense of “my-ness,” “mineness,” “for-me-ness,” or *Meinhaftigkeit* (Sass and Parnas, 2003; Zahavi, 2003; Schneider, 2007). This perspective allows individuals to experience events from a first-personal perspective, recognizing themselves as the subjects of their experiences (Sass and Parnas, 2003; Zahavi, 2003; Parnas and Henriksen, 2014; Henriksen et al., 2019). For instance, when performing an action, having a thought or experiencing emotions, there is an unquestionable recognition that it is indeed oneself, who is acting, thinking and feeling. This implicit, pre-reflective awareness, referred to as the “minimal self” (also termed the “core self,” “basic self,” or *ipseity*—from *ipse*, the Latin word for “self”), imbues all experiences with a sense of “my-ness,” affirming that these experiences belong to the individual (Sass and Parnas, 2003; Parnas and Henriksen, 2019). The minimal self captures, in essence, the experiential sense of being a coherent, living agent—distinct from others and the environment—who maintains a continuous identity over time and is the source of their own thoughts, feelings, and actions (Jaspers, 1920; Casey and Kelly, 2019; Oyebode, 2022). Blanke and Metzinger further refine this into the concept of “minimal phenomenal selfhood,” defined as the most basic form of self-consciousness. Its key features include global identification with one's body, a first-person experiential perspective, and the spatial-temporal localization of the body (Blanke and Metzinger, 2009; Metzinger, 2013).

Self-disorders refer to disruptions in the inner experiences of individuals, particularly affecting the minimal self. Empirical evidence supports the longstanding clinical observation that schizophrenia is characterized by disturbances in the minimal self (Scharfetter, 1981, 2003, 2010; Parnas and Henriksen, 2014). These disturbances, which constitute the “clinical core” of schizophrenia (Sass and Parnas, 2003), are typically non-psychotic phenomena that often emerge in childhood or adolescence, preceding the onset of full-blown psychotic symptoms (Parnas, 2011). To address this core feature of schizophrenia, the Examination of Anomalous Self-Experience (*EASE*) scale was developed in 2005 as a semi-structured clinical tool designed to capture disturbances of the minimal self (Parnas et al., 2005). The *EASE* comprises 57 items grouped into five domains: cognition and stream of consciousness, self-awareness and presence, bodily experiences, demarcation and transitivism, and existential reorientation (Table 1). Unlike broader tools such as the Ego Pathology Inventory (Scharfetter, 2003), which addresses both psychotic and non-psychotic self-experiences, the *EASE* focuses specifically on near-psychotic or non-psychotic phenomena. Self-disorders exhibit a trait-like quality (Henriksen et al., 2021), are more prevalent in schizophrenia spectrum disorders than in other mental illnesses (Raballo et al., 2021; Burgin et al., 2022), demonstrate temporal stability (Nordgaard et al., 2018; Henriksen et al., 2021) and serve as predictors for the development of full-blown psychosis (Henriksen et al., 2021).

**Table 1:** Selected Elements from the Examination of Anomalous Self-Experience (*EASE*) scale, adapted from Parnas et al. (2005).

**Domains**	**Characteristic experiences**
Cognition and stream of consciousness	Diminished sense of thought ownership, externalization of inner speech, intermittent awareness of one's own actions.
Self-awareness and presence	Disruptions in basic selfhood, such as disrupted first-person perspective, inner division, self-alienation, weakened sense of presence, uncertainty about identity, altered experience of age or gender.
Bodily experiences	Changes in perceived body shape, anomalous mirror experiences, estrangement from the body, split between mental and physical self, sensations of disintegration, objectified or spatialized awareness of the body.
Demarcation/transitivism	Blurring of self–other boundaries, confusion with one's mirror image, threatening bodily proximity, sensations of fusion with another.
Existential reorientation	Intensified self-reference, sense of centrality, impression that only one's own experiential world is real, that external reality is illusory, deceptive, or unreal, at times accompanied by solipsistic ideas.

### 5.2 Culture and the minimal self

Culture influences self-representation (Han et al., 2013). A well-documented distinction in this regard is the Independent Self-Construal, which emphasizes autonomy and personal goals (common in individualistic cultures), and the Interdependent Self-Construal, which emphasizes connectedness to others and group harmony (common in collectivist cultures) (Markus and Kitayama, 1991). In turn, the self shapes the culture that accommodates it by reinforcing or modifying norms through daily interactions (Markus and Kitayama, 2010). Culture also appears to affect self-representation at the neural level. For instance, Zhu et al. (2007) demonstrated that medial prefrontal cortex (MPFC) activation differs by culture: in Western cultures, the MPFC responds more selectively to the self, whereas in East Asian cultures, it responds similarly to both the self and close others (Zhu et al., 2007). However, these findings primarily focus on what phenomenologists call the narrative self—the dimension of selfhood involving autobiographical memory, personal identity, and social interactions, shaping how an individual perceives themselves over time (Zahavi, 2010; Horváth, 2024). The minimal self serves as a foundation for the narrative self and also appears to be permeable to cultural influences, at least in some of its dimensions. For instance, while self-other boundary disruptions in Western cultures are typically individualized, affecting internal mental space (e.g., “The government is putting thoughts in my head” or “Aliens implanted a chip in my head and now control my actions”), in Jaffna's sociocentric cultural context, boundary invasiveness is experienced as a relational disorder—one that disrupts not only the individual's sense of self but also family and community structures (Alphonsus et al., 2023).

### 5.3 Enzymatic computation as a framework for self-disorders

In Barrett's enzymatic model of cognition (Barrett, 2005, 2015), thinking is compared to catalysis performed by cognitive enzymes, or “cogzymes,” which process information in a manner similar to how enzymes function within a cell's cytoplasm. A cogzyme acts on a specific chunk of information (its substrate) and then releases the processed output back into a central information pool, making it available for further processing by other cogzymes. This sequential processing resembles a metabolic pathway, such as glycolysis or the Krebs cycle, where the product of one enzymatic reaction serves as the substrate for the next. Just as glycolytic enzymes preserve the energy potential of glucose, cogzymes must retain the “aboutness” of information to ensure its adaptive relevance. For instance, if incoming information signals the presence of a lion, it may be assigned a “LION” tag. Specialized cogzymes can then recognize this tag in a key-and-lock fashion, ensuring that the information is efficiently accessed by the appropriate processing pathways to address the adaptive problem (Barrett, 2005, 2015). Roser and Gazzaniga (2004) and Gazzaniga (2012)'s concept of the *interpreter* provides another perspective, positing a specialized cognitive process that unifies conscious experience by synthesizing the outputs of thousands of information-processing modules operating simultaneously. In this view, Gazzaniga (2012, 2018) argues that conscious experience emerges from the coordinated activity of individual modules rather than from a single, central mechanism. Even in cases of brain damage, such as stroke or dementia, consciousness persists, albeit in a constrained form, as the loss of specific modules limits its scope (Gazzaniga, 2018). Within this framework, the interpreter can be seen as another type of cogzyme, integrating and synthesizing modular outputs into a coherent conscious narrative. However, when the interpreter receives incomplete or erroneous input, the cohesion of conscious experience breaks down, following the familiar principle of “garbage in, garbage out” (Gazzaniga, 2012).

We propose that the pre-reflective sense of ipseity depends on tagging processes within the mind and is therefore vulnerable to their disruptions. Representations may indeed be assigned a “mineness” tag before entering conscious awareness, enabling the interpreter to integrate them into a cohesive product of conscious experience. In the absence of a mineness tag, the interpreter would no longer recognize and integrate these representations. As a result, conscious awareness would receive a disjointed collection of modular outputs, leading to fractured and alien experiences, as captured in the Cognition and Stream of Consciousness domain of the *EASE* scale (Parnas et al., 2005), where thoughts and internal representations are perceived as strange, anonymized, impersonal, and devoid of their sense of belonging. Conversely, overactive tagging may oversaturate consciousness with self-referentiality, producing solipsistic experiences in which the world feels entirely centered on the self, and the subject's perceptions dominate as the only existing reality, as described in the Existential Reorientation domain of the *EASE* scale (Parnas et al., 2005).

A similar tagging mechanism may also apply to bodily representations. If the “global bodily self-identification” tag is absent, the interpreter cannot integrate these representations into a coherent sense of bodily unity. Consequently, the subjective experience of the body becomes fragmented, leading to phenomena like bodily estrangement, morphological alterations, or even sensations of bodily disintegration, producing, in a sense, the sensation of a “disembodied” mind (Fuchs, 2005a). These disruptions align with the Bodily Experiences domain of the *EASE* scale (Parnas et al., 2005). In contrast, melancholic depression, may involve an overactive tagging mechanism that indiscriminately marks body-specific representations. This “over-tagging” results in an overwhelming bodily awareness, leading to what Fuchs (Fuchs, 2005a,b) describes as a “corporealized” mind—a state in which the individual becomes excessively absorbed by their physical body, experiencing a collapse into its spatial and material boundaries (Fuchs, 2005b). Here, the lived body—the experiential medium through which one inhabits the world (*Leib*, or “the body that I am”)—is reduced to an anatomical body (*Körper*, or “the body that I have”) (Fuchs, 2005b). Correspondingly, failure in the “spatiotemporal location” tag may impair the ability to distinguish one's body from external objects or others, leading to disturbances such as mistaking one's mirror image or another person for oneself. This aligns with the Demarcation/Transitivism domain of the *EASE* scale (Parnas et al., 2005). These bodily tagging mechanisms share conceptual similarities with Damasio's somatic marker hypothesis, which suggests that emotionally charged bodily states (“gut feelings”) influence decision-making by steering individuals toward beneficial or detrimental outcomes (Damasio et al., 1996; Damasio, 2000, 2006). Just as somatic markers bias which options become consciously prioritized, embodiment tags may play a crucial role in shaping self-experience by selecting and reinforcing specific bodily representations as central to the sense of self, thereby contributing to its coherence. A breakdown in multiple tagging systems—whether simultaneously or in varying combinations—can result in overlapping anomalies in self-experience, as documented in the Self-Awareness and Presence domain of the *EASE* scale (Parnas et al., 2005).

In conclusion, our proposed model of ego-pathology suggests that cognitive module outputs lose coherence as they enter conscious awareness, leading to the fragmented self often described in self-disorders. Fundamental dimensions of minimal phenomenal selfhood—such as first-person perspective (with its sense of mineness), global bodily self-identification, and spatiotemporal self-location (Blanke and Metzinger, 2009; Metzinger, 2013)—may act as tags that structure conscious self-experience. When these dimensions become disrupted, the individual's sense of agency and coherence erodes, contributing to self-disorders (Table 2).

**Table 2:** Narrative accounts of patients with self-disorders.

**Patient statements**	**Source**
A patient reported having “dense and encapsulated thoughts.”	(Parnas and Handest, 2003)
Referring to trains of thought: “I can get very insecure because their acoustic quality is identical with the quality of a voice of someone standing next to me... It feels as if these (…) aren't really my own.”	(Henriksen and Nordgaard, 2016; p. 267)
“I have been multiplied.”	(Scharfetter, 2010, p. 91, original in German)
“I am us.”	(Scharfetter, 2010, p. 91, original in German)
“I am split into pieces.”	(Scharfetter, 2003, p. 275)
“She splits up into two parts and flies away.”	(Parnas et al., 2005)
“My first-person perspective is replaced by a third-person perspective.”	(Parnas et al., 2005)
“I have the experience that there are two of me: the one that interacts with someone and then there is the real me, who sits there behind.”	(Henriksen and Nordgaard, 2016, p. 267)
“I have a strange feeling that it's somebody else's body.”	(Parnas et al., 2005)
The body feels alien, “as if it did not hang together.”	(Parnas et al., 2005)
“My soul was taken away and distributed.”	(Scharfetter, 2003, p. 275)
“She feels herself as a cranium with something inside, ‘a little man in a cockpit', as if she had two brains.”	(Parnas et al., 2005)
The feeling “as if he was a thing, a refrigerator, and not a human subject.”	(Parnas et al., 2005)
“On the right side I am my father, on the left my mother—and on the nose there is the skin of a cow.”	(Scharfetter, 2003, p. 275)

## 6 Evidence for modularity in self-disorders

### 6.1 Split-brains

At a very broad level, evidence for modularity in the brain comes from patients with disrupted interhemispheric communication, where resulting independent cognitive and motor behaviors for each hemisphere challenge the idea of a unitary self (Gazzaniga, 1977, 2005; Gazzaniga and LeDoux, 1978; Volz and Gazzaniga, 2017). Abnormalities in interhemispheric communication have also been documented in schizophrenia (Foong et al., 2000; Kubicki et al., 2008; Patel et al., 2011; Wang et al., 2024), a phenomenon that may underlie the emergence of auditory hallucinations in the psychosis spectrum (Francis et al., 2016; McGuire, 2016). These observations align with Frith's impaired self-monitoring model, which posits that internally generated thoughts are misattributed to an external source, leading to auditory hallucinations (Frith and Done, 1988; Frith, 2014; chap. 7). Given the functional specialization of the cerebral hemispheres, this loss of interhemispheric synchronization may result in internally generated dialogue in the left hemisphere failing to be recognized as self-produced by the right hemisphere (Frith, 2014, chap. 7), mirroring the cognitive disintegration seen in split-brain patients.

### 6.2 Schizophrenia as dysmodularity

Although hemispheres cannot be strictly considered modules, schizophrenia provides further evidence for dysconnectivity, as it is characterized by widespread white matter abnormalities rather than isolated regional deficits (Kelly et al., 2018). Similar disruptions appear in functional connectivity, where large-scale networks fail to maintain balanced co-activation patterns across brain regions (Liang et al., 2006; Li et al., 2017). This aligns with the “disconnection hypothesis,” which posits that impaired communication between functional networks underlies core symptoms of the disorder (Friston and Frith, 1995). Martin et al. (2023) describe schizophrenic self-disorders as emerging from dysconnectivity both within and between large-scale brain networks, particularly the Salience Network (SN), Default Mode Network (DMN), and Fronto-Parietal Network (FPN), linking these disruptions to the subjective experience of affected individuals (Martin et al., 2023).

The SN, crucial for detecting relevant stimuli and signaling shifts in attention, is hyperactive, leading to excessive self-focus, and an increased awareness of previously filtered-out self-signals. The DMN, involved in self-referential thought and introspection, becomes dysregulated, contributing to disturbances in self-experience and altered self-perception. Meanwhile, the FPN, which regulates executive control, shows disrupted connectivity with the DMN, potentially impairing cognitive regulation over self-monitoring and contributing to misattributions of self-generated thoughts and actions (Martin et al., 2023). This model suggests that a hyperactive SN contributes to excessive self-focus and hyper-reflexivity, heightened SN-DMN connectivity disrupts self-boundaries and impairs the distinction between internal and external stimuli, and reduced DMN-FPN connectivity weakens self-monitoring, collectively driving self-disorders in schizophrenia (Martin et al., 2023). Notably, these alterations are detectable even in unmedicated first-episode psychotic patients, reinforcing that they are intrinsic to the disorder rather than secondary to chronicity or medication effects (Li et al., 2018; Cui et al., 2019). Given that self-disorders often precede full-blown psychosis (Nordgaard and Parnas, 2014), similar network dysfunctions likely occur in pre-psychotic individuals, highlighting their potential as biomarkers for early intervention. This triple-network model suggests that schizophrenic self-disorders involves both reduced network modularity—where specialized networks become less distinct—and the abnormal dominance of certain networks over others.

Considering these findings collectively, self-disorders can be viewed as manifestations of “dysmodularity,” to borrow a term from David (1994), thereby lending renewed attention to the classical Fodorian notion of information encapsulation. From this perspective, self-disorders may metaphorically reflect a modular mind whose encapsulation has been compromised, in which mental modules “decapsulate,” as it were, exposing their internal operations both to each other and to conscious awareness in an unfiltered and often disruptive manner (see also Section 8).

### 6.3 Dissolution of the ego under psychedelics

Dysmodularity and a fragmented self can also manifest in otherwise healthy brains, particularly in states such as psychedelic-induced ego dissolution. This phenomenon shares overlapping neural substrates and exhibits remarkably similar phenomenology with self-disorders (Nour and Carhart-Harris, 2017; Millière et al., 2018). For instance, both psychedelics and self-disorders involve altered information flow within the DMN and SN (Lebedev et al., 2015; Letheby and Gerrans, 2017; Millière et al., 2018; Stoliker et al., 2022). However, while both conditions involve altered large-scale connectivity, their network dynamics diverge. Self-disorders are characterized by rigid, maladaptive hyperconnectivity, whereas psychedelic states promote a more flexible, and globally integrated network architecture, potentially contributing to their therapeutic effects (Carhart-Harris et al., 2013, 2016; Tagliazucchi et al., 2016; Letheby and Gerrans, 2017; Stoliker et al., 2022).

Psychedelics temporarily relax hierarchical constraints, increasing communication between previously segregated brain regions and dissolving functional boundaries (Carhart-Harris et al., 2014; Lebedev et al., 2016; Stoliker et al., 2022). LSD, for instance, enhances global functional connectivity, reducing modularity and strengthening cross-network interactions (Tagliazucchi et al., 2016; Bedford et al., 2023). Crucially, the magnitude of these connectivity changes correlates with participants' subjective ratings of ego dissolution, underscoring the neural basis of self-boundary disintegration (Nour et al., 2016; Tagliazucchi et al., 2016). The phenomenological parallels between these states are equally striking. Individuals may experience loss of self—where personal identity dissolves—disembodiment, derealization, cognitive fragmentation, breakdown of self-other boundaries and fear of dissolution (Hovmand et al., 2024). However, while self-disorders are predominantly characterized by negative emotional connotations, psychedelic experiences can also be mystical, unitive, or even therapeutic (Nour et al., 2016; Letheby and Gerrans, 2017; Stoliker et al., 2022).

Psychedelics shift cognitive processing toward a more associative, non-linear, and emotion-driven mode, mirroring cognitive alterations observed in dreaming and psychosis (Kraehenmann, 2017; Kraehenmann et al., 2017). This cognitive mode, often referred to as primary process thinking and thought to underlie unconscious cognition, stands in contrast to secondary process thinking, which characterizes conscious thought and is inherently logical, structured, and constrained by reality (Brakel, 2004, 2018; Arminjon, 2011; Auchincloss et al., 2012; p. 199–201; Modell, 2014). Under the influence of LSD, participants exhibited hallmark features of primary process cognition, including blurred self-boundaries (“I became part of a metal plate”), surreal transformations (“A cat turned into a wooden clock”), and fluid shape-shifting (“Two people entangled and dissolved into bubbles”) (Kraehenmann et al., 2017). Notably, these effects depended on serotonin 2A receptor (5-HT2A) activation, as their reversal with ketanserin, a 5-HT2A antagonist, confirmed their transient nature (Kraehenmann et al., 2017). These collective findings strongly support the modular mind hypothesis, suggesting that psychedelics disrupt the coordination between distinct cognitive subsystems and, by facilitating the transition between primary and secondary process thinking, may allow their unsynchronized outputs to become consciously accessible. The case of transient, drug-induced ego dissolution, occurring independently of schizophrenia's chronicity, and antipsychotic effects, offers a unique lens into the modular nature of selfhood.

### 6.4 Conclusion of Section 6

Collectively, the phenomenological and neurobiological links between self-disorders and psychedelic-induced ego-dissolution indicate that self-consciousness emerges from the dynamic interplay of multiple components of a modular mind. These findings highlight the fluid and distributed nature of selfhood, challenging traditional views of a unified, monolithic self.

## 7 Empirical approaches to testing the dysmodularity model

To empirically test our model of ego-pathology, where disrupted modular tagging processes expose fragmented modular outputs to conscious awareness, we propose employing guided imagery paradigms in patients with self-disorders. Structured imagery tasks specifically designed to target “mineness,” self-identification, and spatiotemporal coherence could systematically assess subjective experiences of self-fragmentation. Additionally, integrating these paradigms with neuroimaging could reveal the neural correlates of disrupted self-coherence, offering deeper insight into the modular mechanisms underlying selfhood disturbances. A useful organizing principle for this investigation is the distinction between psychological ownership—the sense that one's thoughts and emotions are inherently one's own—and bodily ownership—the sense that one's body and its parts belong to oneself (Bermúdez, 2019).

Assessing mineness or ownership for body parts is relatively straightforward, as patients often spontaneously report deficits in bodily ownership to their examiner. For example, patients with alien limb syndrome would be expected to demonstrate reduced ownership scores when asked, “On a scale from 1 to 10, where 1 represents no sense of belonging and 10 signifies complete ownership, how much does this arm feel like yours?” More severe disturbances of bodily ownership, such as those seen in Cotard Syndrome—where some patients hold the delusional belief that they lack body parts, organs, or biological functions (“I have no organs, no blood, I don't exist physically”) (Berrios and Luque, 1995; Debruyne et al., 2009; Dieguez, 2018)—can be similarly assessed using structured quantitative measures. Beyond bodily ownership, the same methodology can be applied to psychological ownership. Lower scores would be expected in ego-dystonic experiences, such as obsessive thoughts in obsessive-compulsive disorder, intrusive memories in post-traumatic stress disorder, or auditory hallucinations and thought insertion in schizophrenia, where patients perceive these thoughts as originating externally rather than being self-generated. The same quantification methods could systematically assess psychological ownership in patients with self-disorders and may help identify clinically relevant symptom clusters based on individual differences in the intensity of tagging processes.

Subpopulations of self-disordered patients, particularly those experiencing disturbances in bodily experiences as assessed by validation tools (e.g., EASE), could engage in guided motor imagery—externally directed mental simulation of movement without execution (Moran et al., 2012; Hurst and Boe, 2022)—designed to evoke the subjective sense of bodily ownership, provided they do not have aphantasia, the inability to voluntarily generate mental imagery (Zeman et al., 2015; Zeman, 2024). For example, they would be instructed to first imagine their own arm reaching for a cup, and then to imagine a robotic arm performing the same action. Their subjective feelings of mineness would then be assessed using structured quantitative measures, such as “On a scale from 1 to 10, how convinced are you that the imagined arm is yours?” or “How convinced are you that the robotic arm is yours?” The model predicts that patients with disrupted tagging processes should exhibit reduced or altered mineness scores for both conditions, reflecting pre-existing impairments in ownership for their own limb.

Mental imagery tasks, combined with neuroimaging techniques, could be further used to examine neurophenomenological correlations, specifically in the context of self-agency and external attribution, which have been shown to rely on distinct neural systems. The anterior insula, crucial for integrating interoceptive signals and self-generated actions—thereby contributing to the sense of control over one's own movements (Farrer and Frith, 2002; Ohata et al., 2020)—is expected to show atypical activation when individuals with self-disorders imagine their own arm reaching for an object. Such alterations would reflect impairments in sensory-motor integration and self-referential processing. Conversely, when imagining a robotic arm performing the same action, these individuals may exhibit abnormal or undifferentiated activation in the inferior parietal lobule (IPL), a region implicated in differentiating self-initiated from externally generated actions (Farrer and Frith, 2002; Ohata et al., 2020). Disruptions in IPL activation may underlie agency misattribution and weakened self-other boundaries, contributing to experiences of passivity, dissociation, or delusions of control in self-disorders. Notably, similar disturbances in agency processing and self-other differentiation have been experimentally induced in healthy individuals using transcranial magnetic stimulation (TMS) applied to the IPL (Uddin et al., 2006; Chambon et al., 2015), providing an opportunity to systematically investigate parallels between experimental manipulations of agency and the self-disturbances observed in clinical conditions. Moreover, these insights open new avenues for therapeutic intervention. For instance, TMS applied over the temporoparietal junction, a region encompassing parts of the parietal lobe, has led to symptomatic relief in patients with depersonalization disorder (Mantovani et al., 2011). This suggests the potential for targeted neuromodulation strategies to restore agency and self-boundaries in self-disordered patients.

## 8 Information encapsulation

In the classical Fodorian sense, information encapsulation refers to the idea that computations within a module (or inside its “capsule”) are isolated and unaffected by computations in other modules (Fodor, 1983, 2000). While this strict encapsulation may efficiently handle tasks like perception, it fails to account for the flexibility and interactivity required for higher-order cognition. In Barrett's model, cogzymes balance functional specialization with broader informational access. They can broadly access a shared information pool (access generality) but process only inputs matching their specific “binding sites” (processing specificity) (Barrett, 2005; Barrett and Kurzban, 2006). Barrett and Kurzban (2006) further framed encapsulation as a relative concept, arguing that modules operate based on the types or formats of information they can recognize and process. Thus, encapsulation is not absolute but varies depending on the cognitive mechanism and its inputs (Barrett and Kurzban, 2006). Similarly, Carruthers describes encapsulation as a matter of degree. While low-level perceptual systems may exhibit high encapsulation, central systems like reasoning and planning allow for interaction with and querying of other modules. Carruthers distinguishes between wide-scope encapsulation, where most stored mental information does not affect a module's operations, and narrow-scope encapsulation, where modules remain entirely isolated (Carruthers, 2006, chap. 1). This graded view emphasizes how encapsulation supports functional specialization while allowing integration and flexibility, which are essential for human cognition.

In *Why Everyone (Else) Is a Hypocrite* (Kurzban, 2012) and related works (Kurzban and Aktipis, 2006, 2007), Kurzban explains information encapsulation as a default feature of the modular mind, wherein specialized cognitive modules process information independently, ensuring efficiency by focusing on specific tasks without interference. This independence is evident in phenomena like split-brain patients, where distinct modules operate in relative isolation, sometimes producing inconsistencies in thought and behavior. Kurzban links encapsulation to the diversity of representational formats—the ways cognitive modules encode information. Each module processes information in formats optimized for its specific function and may include discursive (propositional, language-like) and iconic (analog, image-like) forms (Quilty-Dunn, 2020). For instance, a visual processing module may rely on iconic representations to encode sensory details, while a social reasoning module may use discursive formats to handle abstract concepts like fairness or intent. These differing representational formats often hinder direct information sharing between modules. Kurzban applies this concept to explain various psychological and behavioral phenomena rooted in the modular mind's independent processing. Cognitive dissonance, self-deception, and moral hypocrisy, for example, arise from the relative isolation of modules and their encapsulated operations, shaped by evolutionary constraints on representational formats and cognitive specialization (Kurzban, 2012). Against this backdrop of encapsulation and diverse representational formats, we will explore intrapsychic conflict in clinical psychiatry.

### 8.1 Clashes between mental modules

The concept of internal conflicts within the mind is far from new. In *Phaedrus*, Plato portrays the soul as consisting of three parts, likened to a chariot pulled by two horses. Reason, depicted as the charioteer, struggles to keep balance between a disciplined horse, representing virtue, and an impulsive one, driven by base desires, whose opposing tendencies makes it difficult to control the chariot (Plato, 2011). Freud similarly conceptualized the mind as tripartite, with the ego (the conscious self) and the superego (morality) attempting to regulate the id (repository of instinctual drives) but they are often dominated by it (Freud, 2019).

Modern theories echo these foundational ideas. Rowan describes semi-independent subpersonalities within the mind, each capable of acting autonomously (Rowan, 1989), while Elliott and Greenberg highlight the “dialogues between aspects of the self” often encountered in therapy (Elliott and Greenberg, 1997). In clinical contexts, such conflicts are vividly observed. For instance, patients with borderline personality disorder often oscillate between wanting to live and wanting to die. Linehan (1993), the founder of dialectical behavior therapy, describes this as a dialectical dilemma: one part wishes to end their life, while another part wants to survive. The therapist's role is to help reconcile these opposing desires. A similar approach is used in motivational interviewing, where therapists address ambivalence between conflicting behaviors, such as wanting to quit substance use while also wanting to continue (Rollnick and Miller, 1995). Obsessive-compulsive disorder further illustrates internal conflict through repetitive behaviors. For instance, while one part of the individual knows the car is locked, another part persistently doubts it, driving compulsive checking. Similarly, in psychosis, ambivalence may manifest as indecision, with patients vacillating for hours or days over seemingly simple choices, such as whether to remain in treatment or leave the hospital (Scharfetter, 2010; Payk, 2021). This theme of conflicting impulses extends to catatonia, where patients may exhibit “ambitendency,” a state of being “stuck” in indecisive movement (Bush et al., 1996), a symptom consistently included in most clinical rating scales for the disorder (Sienaert et al., 2011). For example, a patient might freeze midway while bringing a spoon to their mouth, unable to resolve the urge to simultaneously eat and not eat (Scharfetter, 2010).

Gilbert extends the concept of internal conflict to “social mentalities,” parts of the mind exchanging signals of dominance and submission (Gilbert, 2000). This dynamic is evident in self-criticism, where a dominant “bullying” part attacks a submissive part with thoughts like “You're useless” or “How stupid can you be?” Gilbert extends this concept to schizophrenia, suggesting that humiliating voices or thoughts originate from parts of the self perceived as powerful and dominant, while the receiving part feels powerless and submissive. He proposes therapeutic approaches to challenge the dominance of attacking mentalities by counteracting their influence, fostering moral beliefs to reduce self-criticism, or activating caregiving mentalities for support (Gilbert, 2000). These therapeutic strategies align with specific psychotherapeutic modalities employed in the treatment of dissociative identity disorder, formerly known as multiple personality disorder (International Society for the Study of Trauma and Dissociation, 2011). This condition is characterized by the presence of distinct subpersonalities or alters within the same individual, with at least two of them alternating in taking control over the person's behavior, often accompanied by amnesia for the periods during which an alter is in control (Dorahy et al., 2014; American Psychiatric Association, 2022; Khan, 2023). Some alters are considered more dominant, as illustrated in a female patient of the author G.I., where some therapeutic relief was achieved by opening a dialogue between her dominant, most destructive subpersonality and other aspects of her self, with the goal of identity integration. She shared her experiences of living with multiple personality states in the memoir, *I Am Many*, in the German language (Röper and Hagedorn, 2019).

People often desire incompatible outcomes, creating internal conflicts. A central concept of psychodynamic psychotherapy is that many of these internal struggles occur outside conscious awareness. The clash between different parts of the mind with opposing desires can overwhelm us (e.g., Shedler, 2022; Westen and Gabbard, 2002). To manage this stress, the mind deploys defense mechanisms—unconscious and automatic strategies that help us adapt to internal or external stress and mitigate painful affects (Freud, 1984; Vaillant, 1995; Gabbard, 2014; Cabaniss, 2017). When conflicting parts of the mind are inactive or operate at different times, stress remains low (Cabaniss, 2017). The mind may also find compromises, partially satisfying opposing desires (Collective, 2022). However, when clashing parts are active simultaneously, defense mechanisms activate to reduce internal tension (Cabaniss, 2017; Collective, 2022). These mechanisms are fueled by intrapsychic conflict—unconscious clashes between fears or desires (Cabaniss, 2017)—and their outcome is the alleviation of overstimulation or unpleasant affect.

Defense mechanisms are traditionally classified along a spectrum from less adaptive (“immature”) to more adaptive (“mature”) mechanisms (Cabaniss, 2017; Gabbard, 2014). Less adaptive defenses are based on splitting, where positive and negative aspects of people or experiences are kept separate. In contrast, more adaptive defenses rely on repression, an unconscious process that manages unpleasant thoughts and feelings. If an individual develops object constancy—the ability to recognize that people possess both positive and negative traits—they can use repression to tolerate and integrate distressing emotions. However, without this capacity, unpleasant thoughts and emotions tend to be compartmentalized and often perceived as external threats from others. Cabaniss (2017) describes this dynamic in detail. The specific defense mechanisms a person employs depend on their relative costs and benefits to them. Low-cost, high-benefit defenses can preserve functioning, whereas high-cost, low-benefit defenses may impair long-term functioning, even if they provide temporary relief from distress (Collective, 2022). Nesse (1990b) and Nesse and Lloyd (1992) argues that these trade-offs align with two opposing evolutionary strategies for maximizing fitness: short-term strategies that prioritize immediate personal gains, often at the expense of social relationships, and long-term strategies involving sacrifices that foster reciprocity and future benefits. This dichotomy mirrors Freud's classical distinction between the id, which seeks immediate gratification, and the ego/superego, which regulate behavior for long-term goals and social harmony (Nesse, 1990b; Nesse and Lloyd, 1992). Conceptually, these defenses align with fast life-history traits, which prioritize immediate benefits such as impulsivity, short-term mating strategies, and risk-taking behaviors (Del Giudice and Haltigan, 2023; Del Giudice, 2024). While direct empirical studies linking high-cost, low-benefit defenses to life-history strategies are lacking, indirect evidence suggests a plausible connection. Conditions marked by emotional dysregulation and impulsivity, such as borderline personality disorder, exhibit defense mechanisms like splitting and dissociation alongside fast life-history traits (Brüne, 2016). Although the evidence remains indirect, this conceptual overlap suggests that these defenses may extend beyond intrapsychic conflict resolution, potentially reflecting broader behavioral tendencies linked to life history strategy.

## 9 Clinical evidence for the modular mind and the modular framework for classifying mental disorders

In this final section, we examine how various neuropsychiatric syndromes provide evidence for the modular view and, conversely, how this perspective can serve as an organizing principle for classifying mental disorders. The first part of this section examines how dissociation studies support the decomposability of cognitive processes into distinct modular elements. It further considers how lesion studies provide evidence for specific modules proposed by evolutionary psychology, such as the animacy detector, the agency detector and cognitive mechanisms for reasoning about artifacts. In the second part, we consider the potential for organizing neuropsychiatric clinical evidence along modular lines and discuss the implications of using dysfunctional modules as a framework for classifying neuropsychiatric illness.

### 9.1 Supporting the modular view through clinical evidence

Clinically recognized disorders, such as those discussed in Sections 5, 6, 8, provide compelling empirical support for the existence of mental modules. Dissociation studies have historically highlighted a modular architecture of the mind, demonstrating that distinct neuropsychological functions can be selectively impaired while others remain relatively intact (Baddeley, 2003; Caramazza and Coltheart, 2006; Dunn and Kirsner, 2003; Gerlach et al., 2018; Machery, 2012; but see Plaut, 1995). For example, in melancholia and personality disorders, it is the narrative self—shaped by identity, life stories, and beliefs—that is disrupted, while the minimal self, which is affected in schizophrenia spectrum disorders, remains intact (Parnas and Henriksen, 2019). A clear dissociation between knowledge domains is evident in cases where knowledge about the functions of tools remains distinct from knowledge about how to manipulate them (Johnson-Frey, 2004; Boronat et al., 2005; Garcea and Mahon, 2012; Garcea et al., 2013). For instance, patients with apraxia—a condition characterized by an inability to perform complex learned actions (Heilman, 2010; Berkowitz, 2022)—exhibit selective impairments in manipulation knowledge while retaining the ability to recognize the function of objects (Buxbaum et al., 2000; Buxbaum and Saffran, 2002). Similarly, vision and action can be dissociated in specific deficits affecting these components (Goodale et al., 1994). Patients with optic ataxia, such as patient RV, can “see but cannot grasp”—they are unable to reach for or grasp objects despite being able to visually perceive them (Perenin and Vighetto, 1988; Goodale et al., 1994; Schindler et al., 2004; Karnath and Perenin, 2005). Conversely, cases like patient DF demonstrate the opposite dissociation—being able to “grasp but not see” (Goodale et al., 1991, 1994; Milner et al., 1991; James et al., 2003). Dissociation studies also demonstrated significant sharing in processing mechanisms, as it is rare for a module to be affected without neighboring ones also experiencing some degree of impairment. For example, in the ventral temporal cortex, representations of objects and faces overlap substantially (Haxby et al., 2001). Lesion studies reveal that focal damage in this area can result in prosopagnosia, while more extensive damage often leads to broader difficulties in object recognition (Damasio et al., 1982; Farah, 1991; Gauthier et al., 2000). By the same token, disruptions in the minimal self in schizophrenia can also lead to instability in the narrative self, as the former serves as a precondition for the latter (Scharfetter, 2010, chap. 3; Parnas and Henriksen, 2019). In summary, dissociations offer robust clinical evidence for the modularity of distinct neuropsychological systems, while simultaneously recognizing their interconnectedness.

Not only does clinical evidence from neuropsychiatric syndromes support the decomposability of cognitive processes into distinct modules, but it may also help identify new modular elements. For instance, conditions such as akinetopsia (the inability to perceive motion) (Zihl et al., 1983; Stevens et al., 2009) and kinetopsia (perceiving stationary objects as moving) (Blom, 2023), observed in various neuropsychiatric syndromes (Browne et al., 2024)—including Alice in Wonderland Syndrome, which involves distortions in visual perception, body schema, and the experience of time (Blom, 2016)—suggest the existence of an animacy detector. This mechanism classifies objects as animate or inanimate (Leslie, 1994; Scholl and Tremoulet, 2000; Barrett, 2015). While motion itself takes various forms (Park and Tadin, 2018), biological motion, characterized by self-propulsion, goal-directed behavior, and responsiveness to external events (Barrett, 2015, p. 108), is distinct from mechanical motion. These mechanisms, referred to as “life detectors” (Troje and Westhoff, 2006), are crucial for distinguishing animate from inanimate entities. Anomalies in perceiving biological motion have been documented in disorders such as schizophrenia (Kim et al., 2005, 2013) and autism spectrum disorders (Freitag et al., 2008; Todorova et al., 2019; Federici et al., 2020).

In self-disorders, disturbances in the sense of agency—the perception of oneself as the initiator and controller of thoughts, feelings, and actions—are also striking (Scharfetter, 1981, 2003; Gallagher and Trigg, 2016). Patients may phenomenologically experience their actions or thoughts as being externally produced or manipulated, a phenomenon referred to as delusions of control (American Psychiatric Association, 2022). These patients often describe having implanted devices, purportedly used for surveillance or control of their bodily functions (e.g., “They inserted a computer chip in my brain to control me”). This clinical phenomenon supports the idea of a “hypersensitive agent detection device,” an adaptive mechanism that predisposes humans to perceive agency and intentionality, even in improbable situations (Barrett, 2004). This bias likely evolved as an error-management strategy, enhancing survival by erring on the side of caution—e.g., interpreting rustling leaves as a potential predator (Barrett, 2004; Douglas, 2020). When these delusions specifically involve artifacts, they align with Dennett's concept of the “design stance,” which ascribes intention to an actual or presumed creator when interpreting an artifact (Dennett, 1990). Additionally, this reflects a universal cognitive tendency across cultures: reasoning about artifacts often focuses on the original intentions behind their creation (German and Johnson, 2002; Defeyter and German, 2003; Kelemen and Carey, 2007; Barrett et al., 2008). In delusions of control, patients may interpret the “implanted” artifacts as instruments of malicious intent.

### 9.2 Organizing clinical evidence through a modular lens

Several authors have advocated for using evolved cognitive mechanisms as the basis for classifying mental conditions (Wakefield, 1992, 2015; Cosmides and Tooby, 1999; Murphy and Stich, 2000; Zielasek and Gaebel, 2008, 2009; Giudice and Ellis, 2016; Del Giudice, 2018). Such a classification system holds significant promise, but the initial step in this endeavor requires a comprehensive cataloging of the human mind and a detailed analysis of how evolved mental modules may fail or become compromised. Nesse provides valuable insights into why these systems are inherently vulnerable to breakdown (Nesse, 2005, 2015; Nesse, 2019).

Efforts to identify evolved mental modules are already ongoing, though debates persist about what constitutes an evolved mental module and how best to “carve the mind at its joints.” A central challenge is the “grain problem,” which concerns defining the number and scope of distinct adaptive problems and their corresponding mental modules (Atkinson and Wheeler, 2003). Addressing this issue and the broader lack of consensus across behavioral science may require a cross-disciplinary approach (Brase, 2014). Such an approach could provide converging evidence from independent sources, fostering a more unified understanding of the mind's modular nature—an aim long championed by evolutionary psychology (e.g., Al-Shawaf et al., 2020; Buss, 2019; Lewis et al., 2017; Tooby and Cosmides, 1992). Schmitt and Pilcher (2004) propose a heuristic framework for integrating and evaluating evidence of psychological adaptations across disciplines. Their model assesses psychological features from eight evidentiary sources: theoretical, psychological, medical, physiological, genetic, phylogenetic, hunter-gatherer, and cross-cultural data. Evidence is then evaluated based on evidentiary breadth (the number of supporting disciplines) and evidentiary depth (the rigor of the research) (Schmitt and Pilcher, 2004). Building on this framework, Balachandran and Glass developed PsychTable (https://app.psychtable.org/), “a collaborative web-based project aimed at classifying and evaluating evolved psychological adaptations (EPAs).” PsychTable systematically organizes EPAs, serving as a classification system akin to the Periodic Table of Elements or *Gray's Anatomy* (Balachandran, 2011; Balachandran and Glass, 2012; Glass and Balachandran, 2020).

Aligning with this framework, each module in such a classificatory system would ideally include a detailed description of its functions and subcomponents, developmental trajectory, evolutionary homologies with other species, and cultural variations. For example, in the case of Mindreading, humans appear uniquely capable of consistently passing the false-belief task (Call and Tomasello, 2008), an ability that typically develops around the age of four (Wellman et al., 2001) and is universal across cultures (Onishi and Baillargeon, 2005; Barrett et al., 2013). However, the ability to track other types of mental states is not exclusive to humans. It is also observed in non-human primates (Call and Tomasello, 2008), canids (Hare et al., 2002), and corvids (Clayton et al., 2007) (reviewed in Barrett, 2020a, 2015). Beyond false-belief tasks, mindreading also involves shared intentionality and triadic representations (Barrett, 2020a)—where two individuals simultaneously focus on an external entity (Tomasello et al., 2005; Saxe, 2006; Tomasello, 2019)—as well as unique forms of empathy (Decety and Jackson, 2004; Saxe, 2006; Decety and Svetlova, 2012).

The folkbiological system (FBS) (Medin and Atran, 2004) reflects a universal human tendency, observed across cultures, to classify the living world into ranked, species-like taxonomies while attributing an “essence” to organisms—intrinsic, immutable properties inherited from their parents that determine their observable characteristics (Atran, 1998, 1999, 2002; Medin and Atran, 2004). The FBS shows notable differences between modern industrialized societies and non-WEIRD societies. For instance, children in industrialized societies often categorize humans as separate from animals earlier in life, while children in non-WEIRD societies are more likely to see humans as another type of animal at a similar developmental stage (Medin et al., 2010). Additionally, both children and adults in non-industrialized societies are more inclined to emphasize ecological relationships between living beings compared to their counterparts in majority-culture societies (Ross et al., 2003; Medin et al., 2006; Bang et al., 2007). A compelling example of evolved cognitive strategies within FBS is the Plant Learning and Avoidance of Natural Toxins (PLANT) system, which comprises behavioral strategies and social learning rules for reasoning about plants (Wertz, 2019). Infants exhibit a clear avoidance response, demonstrating reluctance to touch plants compared to other objects (Wertz and Wynn, 2014). Toxic plant avoidance is also observed in non-human animals, demonstrating evolved strategies to mitigate the risks associated with plant defenses. For instance, herbivores employ mechanisms like post-ingestive feedback, as seen in sheep, which learn to avoid harmful plants through negative physiological experiences (Provenza, 1995; Karban and Agrawal, 2002). In humans, this response manifests as food neophobia, the reluctance or fear of trying new foods (Pliner and Hobden, 1992; Pliner and Salvy, 2006). This tendency is particularly pronounced for plant-based foods, likely as an adaptive mechanism to protect infants from ingesting plant toxins once they become mobile and capable of independently selecting food (Cashdan, 1998; Wertz and Wynn, 2014, 2019; Włodarczyk et al., 2018). Evidence for this mechanism comes from the inverse relationship between food neophobia and vegetable consumption in both children (Johnson et al., 2015; Xi et al., 2022; Estay et al., 2023) and adults (Jaeger et al., 2017; Costa et al., 2020) and from the observation that neophobia scores increase between ages 1 and 2 across different cultural backgrounds, coinciding with the period when infants begin to gain mobility (Estay et al., 2023).

Building on the concept of dissociation, it is possible to map modules and their subcomponents onto the corresponding disruptions observed in various neuropsychiatric disorders. In some cases, clinically relevant dysfunctions align closely with specific modular failures, occasionally in a one-to-one correspondence. For instance, within the intuitive mechanics module, deficits often align along the what/where distinction (Putcha et al., 2021). Impairments in tracking the identity of objects manifest as an inability to recognize objects (Farah, 2004), familiar places (Landis et al., 1986), faces (Kanwisher et al., 1997; Kanwisher and Yovel, 2006), or text (Cohen et al., 2000). Spatiotemporal tracking deficits include topographical disorientation, where patients struggle to use landmarks for visuospatial navigation (Aguirre and D'Esposito, 1999; Erkkinen et al., 2021), and difficulty perceiving the motion of objects (Zihl et al., 1983; Stevens et al., 2009). Some lesions affect entire modular subcomponents, as seen in the case of patient RS. Following an ischemic stroke affecting the left posterior cerebral artery, patient RS exhibited a disproportionate semantic impairment specifically for fruits and vegetables, while knowledge of animals and artifacts remained relatively preserved (Samson and Pillon, 2003). Dyscalculia offers another example, resulting from impairment to the “number sense,” defined as the “ability to quickly understand, approximate, and manipulate numerical quantities” (Dehaene, 2001). Additionally, lesions can affect distinctions between animate and inanimate entities. Brain injuries frequently result in disproportionately severe impairments in recognizing and reasoning about living things compared to inanimate objects (Caramazza and Shelton, 1998; Forde, 1999; Humphreys and Forde, 2001; Tyler and Moss, 2001; Capitani et al., 2003; Mahon and Caramazza, 2009). This distinction is fundamental, present from early development in humans (Greif et al., 2006), and observed even in blind individuals (Mahon et al., 2009). Knowledge about animals and plants holds a privileged status in the brain due to its critical role in human survival—animals as predators or sources of food, plants as food or toxin-producers—with their specialized yet vulnerable neural representation reflected in the disproportionate impairment of these biological categories in semantic deficits (Caramazza and Shelton, 1998; Capitani et al., 2003).

Classical nosologic syndromes also exhibit disruptions in shared modular components. For example, studies of autistic children consistently show significant impairments in passing the false-belief task, despite intact capacities in non-mentalistic tasks (Baron-Cohen et al., 1985; Baron-Cohen, 1995, 1997). However, mindreading involves more than false-belief reasoning as already seen. It includes components like shared attention mechanisms (Baron-Cohen, 1997; Tomasello et al., 2005; Tomasello, 2019) and gaze detection mechanisms (Baron-Cohen, 1995, 1997). Autistic children often exhibit deficits in shared attention (Rogers and Pennington, 1991; Carpenter et al., 2001; Mundy and Newell, 2007), struggle to follow others' gaze in social contexts (Leekam et al., 1997; Nation and Penny, 2008), fail to direct their gaze toward social stimuli (Dawson et al., 1998), and show impairments in initiating joint attention, which involves using eye contact to direct others' attention to an object or event (Mundy, 2003). In schizophrenia, dysfunction spans multiple modules. It has been suggested that schizophrenia represents “the price that Homo sapiens pays for language” (Crow, 1997, 2000, 2008). Indeed, schizophrenia patients exhibit significant language deficits, including difficulties in comprehension (Kuperberg, 2010a; Perlini et al., 2012), higher-order language processing (Kuperberg, 2010b), decreased production of syntactically complex sentences (DeLisi, 2001; Kircher et al., 2005), and impairments in semantics (Goldberg and Weinberger, 2000; Salavera et al., 2013) and pragmatics (Kuperberg et al., 1998; Covington et al., 2005; Perlini et al., 2012; Salavera et al., 2013). Furthermore, they demonstrate significant deficits in mindreading tasks (Brüne, 2005) and struggle to comprehend conceptual metaphors, a phenomenon referred to as concretism (Rossetti et al., 2018). For instance, when asked to interpret the figurative expression “A rolling stone gathers no moss,” patients with schizophrenia might provide a literal interpretation such as “Because the stone is rolling, there's no time for the moss to attach to it.”

The breakdown of a modular system extends beyond semantic impairments, impacting developmental changes and the causal mechanisms underlying biological concepts (Zaitchik and Solomon, 2001). For example, the development of the FBS progresses from an early behavioral theory—focused on motion—to a mature biological theory encompassing life cycles and bodily processes such as respiration, birth, growth, disease, and death (Carey, 1985). Early in development, children exhibit animistic tendencies, categorizing independently moving objects, including inanimate ones like the sun, fire, or cars, as “alive” (Carey, 1985; but also see Barrett and Behne, 2005). During this stage, essentialist thinking—wherein an organism's intrinsic “essence” is seen as immutable—is absent, leading children to believe that an animal's identity could change based on superficial appearance (reviewed in Solomon and Zaitchik, 2012). These developmental phenomena re-emerge in conditions like Alzheimer's disease (AD). Zaitchik and Solomon (2008) documented that AD patients often attribute life to inanimate objects capable of self-generated activity, such as cars or fire, reflecting a regression to animistic thinking (Zaitchik and Solomon, 2008). Furthermore, AD patients demonstrate impaired essentialism, as shown in their performance on Keil's Species Transformation Task (Zaitchik and Solomon, 2009). In this task, about half of the AD patients believed that superficial changes, like painting stripes on a horse to resemble a zebra or dressing a chicken in a turkey costume, could cause the animal to cross species boundaries. These findings underscore how classification along modular lines offers a deeper understanding of the causal principles within a conceptual domain, surpassing simplistic interpretations of semantic impairments.

In some classical psychiatric phenomena, modular elements manifest clinically at the interfaces between different cognitive and perceptual domains. For instance, animals, as a component of the FBS, are implicated in a range of disorders. Examples include animal phobias (Norberg et al., 2024), animal hallucinations (Platz et al., 1995; Fénelon et al., 2000; Ffytche, 2005), and lycanthropy, the delusional belief of transforming into a wolf (Keck et al., 1988; Khalil et al., 2012; Blom, 2014; Guessoum et al., 2021). Other phenomena related to this modular system include zooanthroprosopmetamorphopsia, where individuals perceive human faces morphing into animal faces (Blom et al., 2021; Blom, 2023), Noah's Syndrome (animal hoarding) (Patronek, 1999; Ferreira et al., 2017; Abreu and Marques, 2022; Nadal et al., 2022; Stumpf et al., 2023), delusional parasitosis, characterized by the belief of infestation with crawling pathogens (Trabert, 1995; Freudenmann and Lepping, 2009), and the Dolittle phenomenon, where individuals experience animals speaking (Dening and West, 1990; Hirsch, 2023). In the context of musicality, the cognitive processes underlying music perception and behavior (Honing and Ploeger, 2012; Honing, 2018), examples include musical anhedonia, characterized by an inability to derive pleasure from music despite experiencing normal enjoyment in other areas of life (Mas-Herrero et al., 2014), musical hallucinations, where individuals perceive music that is not externally present (Blom, 2023), and musical obsessions, which involve involuntary and intrusive musical thoughts or tunes that align with the criteria for obsessions (Taylor et al., 2014).

A modular framework can also provide insights into comorbidity between psychiatric disorders. Disorders affecting overlapping modules often share clinical features. Theoretically, if Disorder 1 involves dysfunctions in modules A, B, and C, and Disorder 2 involves dysfunctions in modules C, D, and E, their symptomatology would overlap due to the shared impairment in module C. For example, dysfunctions in mindreading modules are commonly observed in autism spectrum disorders (Baron-Cohen et al., 1985; Baron-Cohen, 1995, 1997, 2000), schizophrenia (Abu-Akel, 1999; Abu-Akel and Bailey, 2000; Brüne, 2005), bipolar disorder, major depressive disorder, social anxiety disorder, eating disorders, and borderline personality disorder (reviewed in de la Higuera-González et al., 2023). These shared dysfunctions help explain overlapping symptoms, such as difficulties in social cognition, across these conditions.

## 10 Discussion

The mind conceived as an assemblage of evolved information-processing modules offers a powerful organizing principle for understanding many phenomena of psychiatric interest, including selfhood disorders, intrapersonal conflict, and even the classification of mental conditions along modular lines. In self-disorders, a dysfunctional minimal self may expose the mind's underlying modular architecture to conscious awareness, prompting a sudden and unsettling recognition of its internal multiplicity. A relaxed interpretation of information encapsulation, combined with the varied ways information is stored across different parts of the mind, could illuminate the inconsistencies in thought and behavior that underpin intrapsychic conflict. Well-defined clinical disorders could help bolster support for proposed mental modules or suggest novel ones. Conversely, mapping the modular framework onto neuropsychiatric syndromes can enhance our parsing of clinical reality and refine our conceptualization of psychiatric illness, enabling a more nuanced recognition of patients' lived experiences and, in turn, deepening our empathic understanding of their suffering. In conclusion, should cognitive modularity persist as a biologically plausible and empirically validated framework, it would resonate profoundly with the core phenomena that psychiatry has grappled with for over two centuries.

## Data Availability

The original contributions presented in the study are included in the article/supplementary material, further inquiries can be directed to the corresponding author.
